# Identification of accession-specific variants and development of KASP markers for assessing the genetic makeup of *Brassica rapa* seeds

**DOI:** 10.1186/s12864-022-08567-9

**Published:** 2022-04-25

**Authors:** Seongmin Hong, Su Ryun Choi, Jihyeong Kim, Young-Min Jeong, Ju-Sang Kim, Chun-Hee Ahn, Suk-Yoon Kwon, Yong Pyo Lim, Ah-Young Shin, Yong-Min Kim

**Affiliations:** 1 Genome Editing Research Center, Korea Research Institute of Bioscience and Biotechnology (KRIBB), Daejeon, Republic of Korea; 2 Molecular Genetics and Genomics Laboratory, Department of Horticulture, College of Agriculture and Life Science, Chungnam National University, Daejeon, Republic of Korea; 3 XENOTYPE Inc., 118 Jungang-ro, Jung-gu, Daejeon, 34912 Republic of Korea; 4 Seed Industry Promotion Center, Foundation of Agri. Tech. Commercialization & Transfer (FACT), Gimje, Republic of Korea; 5 Dayi International Seed Co., Ltd., 16-35 Ssiat-gilJeollabuk-do, Baeksan-myeon, Gimje, 54324 Korea; 6 Plant Systems Engineering Research Center, Korea Research Institute of Bioscience and Biotechnology, Daejeon, Republic of Korea

**Keywords:** Seed purity assessment, KASP marker, Accession-specific marker, *Brassica rapa*, *B. rapa* breeding

## Abstract

**Background:**

Most crop seeds are F1 hybrids. Seed providers and plant breeders must be confident that the seed supplied to growers is of known, and uniform, genetic makeup. This requires maintenance of pure genotypes of the parental lines and testing to ensure the genetic purity of the F1 seed. Traditionally, seed purity has been assessed with a grow-out test (GOT) in the field, a time consuming and costly venture. Early in the last decade, seed testing with molecular markers was introduced as a replacement for GOT, and Kompetitive allele specific PCR (KASP) markers were recognized as promising tools for genetic testing of seeds. However, the markers available at that time could be inaccurate and applicable to only a small number of accessions or varieties due to the limited genetic information and reference genomes available.

**Results:**

We identified 4,925,742 SNPs in 50 accessions of the *Brasscia rapa* core collection. From these, we identified 2,925 SNPs as accession-specific, considering properties of flanking region harboring accession-specific SNPs and genic region conservation among accessions by the Next Generation Sequencing (NGS) analysis. In total, 100 accession-specific markers were developed as accession-specific KASP markers. Based on the results of our validation experiments, the accession-specific markers successfully distinguised individuals from the mixed population including 50 target accessions from *B. rapa* core collection and the outgroup. Additionally, the marker set we developed here discriminated F1 hybrids and their parental lines with distinct clusters.

**Conclusions:**

This study provides efficient methods for developing KASP markers to distinguish individuals from the mixture comprised of breeding lines and germplasms from the resequencing data of Chinese cabbage (*Brassica rapa* spp. *pekinensis*).

**Supplementary Information:**

The online version contains supplementary material available at 10.1186/s12864-022-08567-9.

## Background

Most growers of vegetable crops rely on F1 hybrid seeds, and suppliers of these seeds must maintain genetically pure stocks. Not only do the suppliers need to keep seeds of known genetic makeup for sales but also for their ongoing breeding programs. Until the late 1990s, seed providers relied on what is known as the grow-out test (GOT), in which the seeds were planted in the field and the traits of the test plants were assessed by investigation [1]. However, this method is time consuming, requires a large amount of land, and is partly subjective as plant phenotype can be affected by the environment [2]. Thus, precise and efficient tools to assess the genetic makeup and purity of hybrid seeds are sought by seed providers.

In response to these limitations of the GOT, various types of molecular markers have been developed to characterize the genotypes of crop plants. This endeavor began in the early 1990s and has resulted in the identification of numerous types of markers. These include restriction fragments length polymorphism, amplified fragments length polymorphism, simple sequence length polymorphism, simple sequence repeat (SSR), and sequence tagged site (STS) markers. The PCR-based SSR or STS markers can be rapidly acquired, are easy to assay, and have been used for crop breeding or assessment of hybrid seeds in rice, maize, pigeon pea, and pepper [1–4]. However, these markers were developed for specific breeding lines or varieties and are not sufficient to assess the purity of hybrid seeds.

Application of molecular markers to a wide range of situations that require accurate assessment of the genetic makeup of a plant must entail investigating genetic variants in both the core collections and commercial lines. Previous investigation of genetic variants of core collections and commercial crop lines was limited because of the expense of sequencing and the absence of reference genomes. With the advent of next-generation sequencing technology, reference genomes have been constructed for a number of crops, including tomato [5], pepper [6], cucumber [7], melon [8, 9], wheat [10], and Chinese cabbage [11]. Whole genome resequencing of various crops has also been undertaken. This has allowed the development of widely applicable molecular markers, accomplished by resequencing analyses of core collections. Also, the development of the Kompetitive Allele Specific PCR genotyping (KASP) assay has permitted the development of accession-specific markers for large-scale seed purity assessments [12–14].

Here, we present pipelines for the detection of accession-specific genetic variants and accession-specific markers from 50 Chinese cabbage accessions. The pipelines were constructed with a combination of genetic variants calling, detection of accession-specific variants, and determination KASP marker candidate sequences. Accession-specific single nucleotide polymorphisms (SNPs) were identified from 50 Chinese cabbage core collections, and 100 accession-specific KASP markers from 50 accessions were developed from a pool of these SNPs. Then, evaluation of KASP markers was carried out using the core collection and 35 non-core collections. We have identified 100 KASP markers that we believe will be useful in assessing hybrid seed purity.

## Results

### Identification and evaluation of accession-specific variants

**Fig. 1:**
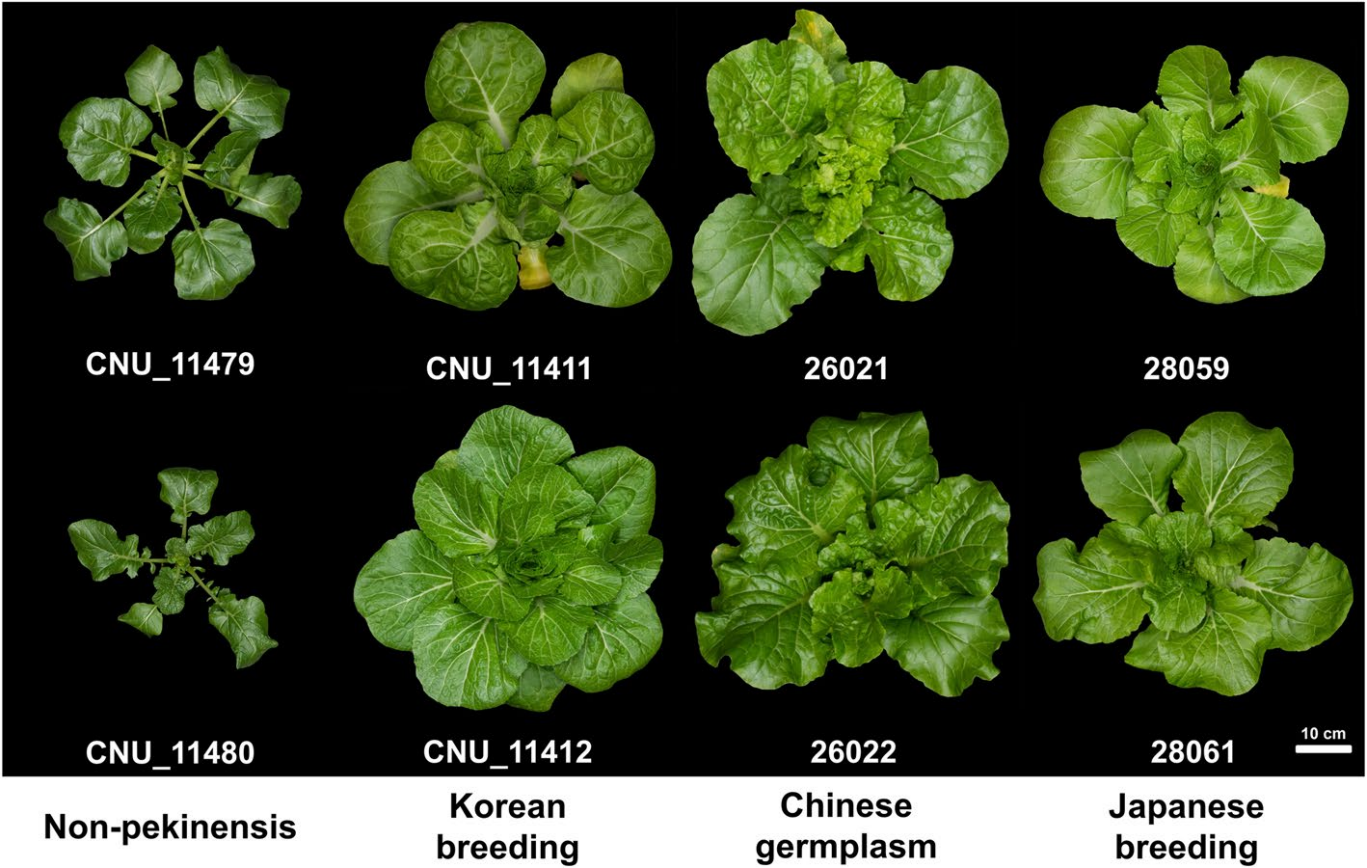
Morphological features of eight representative accessions from four groups of the *Brassica rapa* core collection

**Fig. 2:**
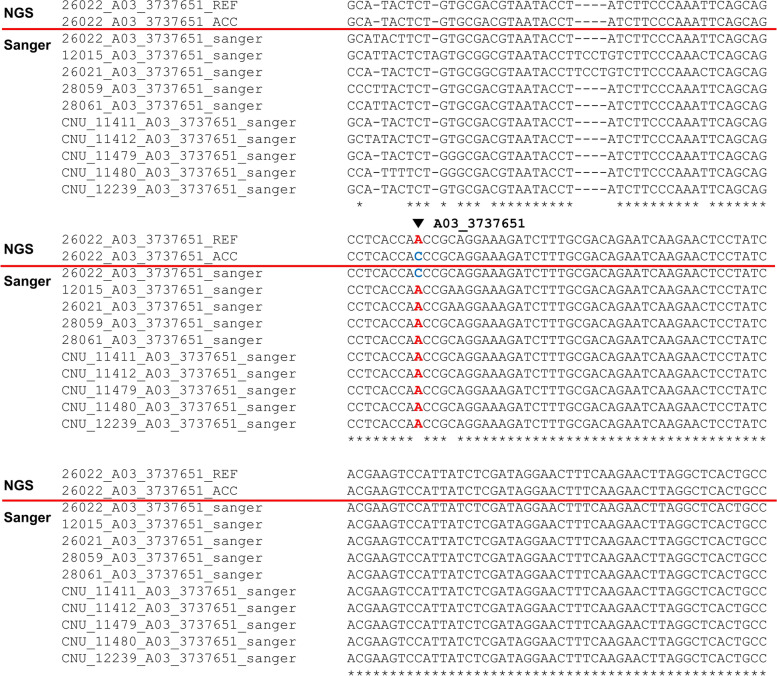
Validation of accession-specific single nucleotide polymorphisms (SNPs) (3,737,651 in chromosome 3) from accession 26,022 (from Chungnam National University) using *the Brassica rapa* reference genome (ver 3.0). (REF, reference genome; ACC, resequencing result of individual accession; Sanger, Sanger sequencing result)

We performed genome resequencing analysis of 50 accessions from the *Brassica rapa* core collection, with the goal of developing markers specific to each accession. This core collection is composed of five groups: non-pekinensis, Chinese, Japanese, Korean breeding lines, and others (Fig. 1 and Supplementary Table 1). We mapped the reads from the analysis of these accessions to the *B. rapa* reference genome (ver 3.0) [11] with the BWA-MEM (ver 0.1.17) using the default parameters. We detected a total of 4,925,742 SNPs from the 50 accessions (Table 1 and Supplementary Data 1). Since we wished to identify genetic variants from the *B. rapa* core collection, we constructed a variant-identification pipeline by combining the calling and filtering variants (Supplementary Fig. 1). This entailed first detecting and merging SNPs of individual accessions in the joint variant calling step. Next, we identified homozygous alternative alleles for single accessions as accession-specific SNPs by comparing the pattern of variants of each individual accession in the core collection. To develop KASP markers, we evaluated each accession-specific marker by considering the non-redundant flanking sequences, overlapping of repeat sequences, and annotation of the SNPs. Finally, we identified SNPs with unique flanking sequences without overlapping repeat sequences as candidates for development of KASP markers. We identified 2,925 accession-specific SNPs as such candidates (Table 1), most of which were located in flanking gene sequences and 2,806 of which (approximately 95.9%), were in genic regions (Table 2). Of these 2,925 candidate SNPs, approximately 456, or 15.6%, resulted in non-synonymous mutations, and 19 variants led to abnormal termination of translation. These genetic variants may be important in future investigation of trait-associated genes or markers. Our next step in the development of accession-specific markers was to validate the SNPs with genome resequencing analysis, which we did with Sanger sequencing (Fig. 2).

**Table 1:** The single nucleotide polymorphisms (SNPs) that were identified from 50 *B. rapa* accessions

Group	Numbers of accessions	Total number of identified SNPs	Total number of accession-specific SNPs
Korean	34	4,095,628	1,314
Chinese	5	3,289,533	357
Japanese	3	2,191,837	325
Non-pekinensis	2	2,016,767	429
Others	6	3,044,951	500
Total	50	4,925,742	2,925

**Table 2:** Annotation of the accession-specific single nucleotide polymorphisms (SNPs) that were identified from the *B. rapa* core collection

Annotation of variants	Type	Accession-specific SNPs (No.)	KASP markers (No.)
Variant causes a codon that produces a different amino acid	Exon	456	72
Variant causes a codon that produces the same amino acid	Exon	189	18
Variant causes a STOP codon	Exon	17	2
Variant causes start codon to be mutated into a non-start codon	Exon	1	0
Variant causes stop codon to be mutated into a non-stop codon	Exon	1	0
Variant causes stop codon to be mutated into another stop codon	Exon	3	1
The variant hits a splice acceptor site	Intron	5	0
The variant hits a Splice donor site	Intron	4	0
Variant hits intron	Intron	122	1
Downstream of a gene (default length: 5 K bases)	Non-coding	459	0
The variant is in an intergenic region	Non-coding	119	0
Upstream of a gene (default length: 5 K bases)	Non-coding	1549	6
Total	-	2,925	100

We select eight flanking sequences of the accession-specific SNP candidates from the four groups of the core collection and Sanger sequencing primers were designed (Supplementary Table 2). From the Sanger sequencing results, we concluded that seven of the SNP candidates were specific to a single accession (Fig. 2 and Supplementary Figs. 2, 3, 4, 5, 6 and 7). Amplification by PCR for Sanger sequencing failed in one flanking sequence (Supplementary Fig. 8), leading us to conclude that SNPs with conserved flanking sequences were the best candidates for developing accession-specific markers with PCR. Also, candidate SNPs with highly conserved flanking sequences that are suitable for primers may be necessary for developing wide-ranging KASP markers that will apply to crops not in the core collection or to commercial cultivars. Determination of primer sites for KASP markers is important for the development of accession-specific KASP markers.

### Development and evaluation of KASP markers

**Fig. 3:**
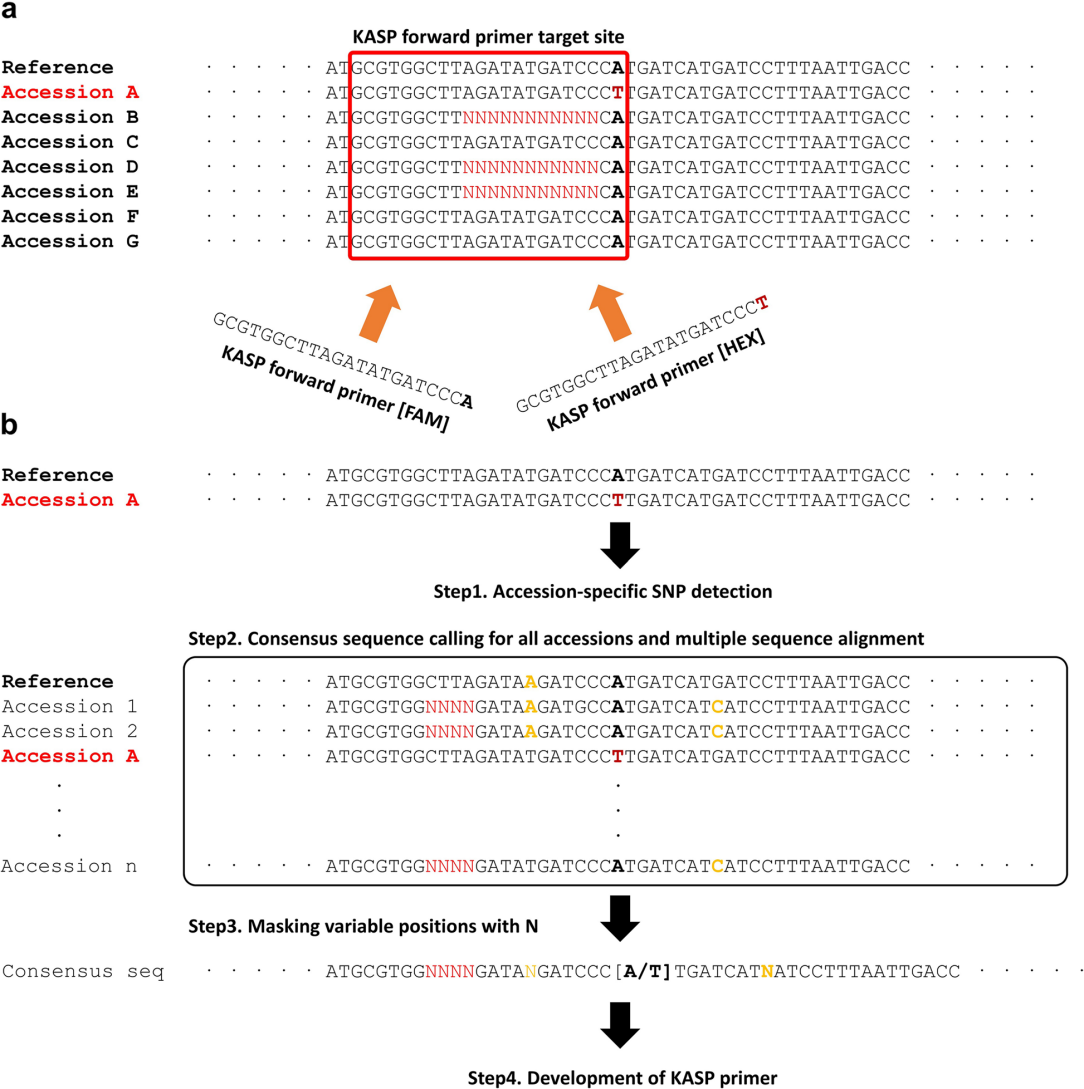
Development of KASP markers. **a** Potential problem of primer alignments by possible sequence variation from core collection during KASP marker development, **b** Process for development of KASP markers

**Fig. 4:**
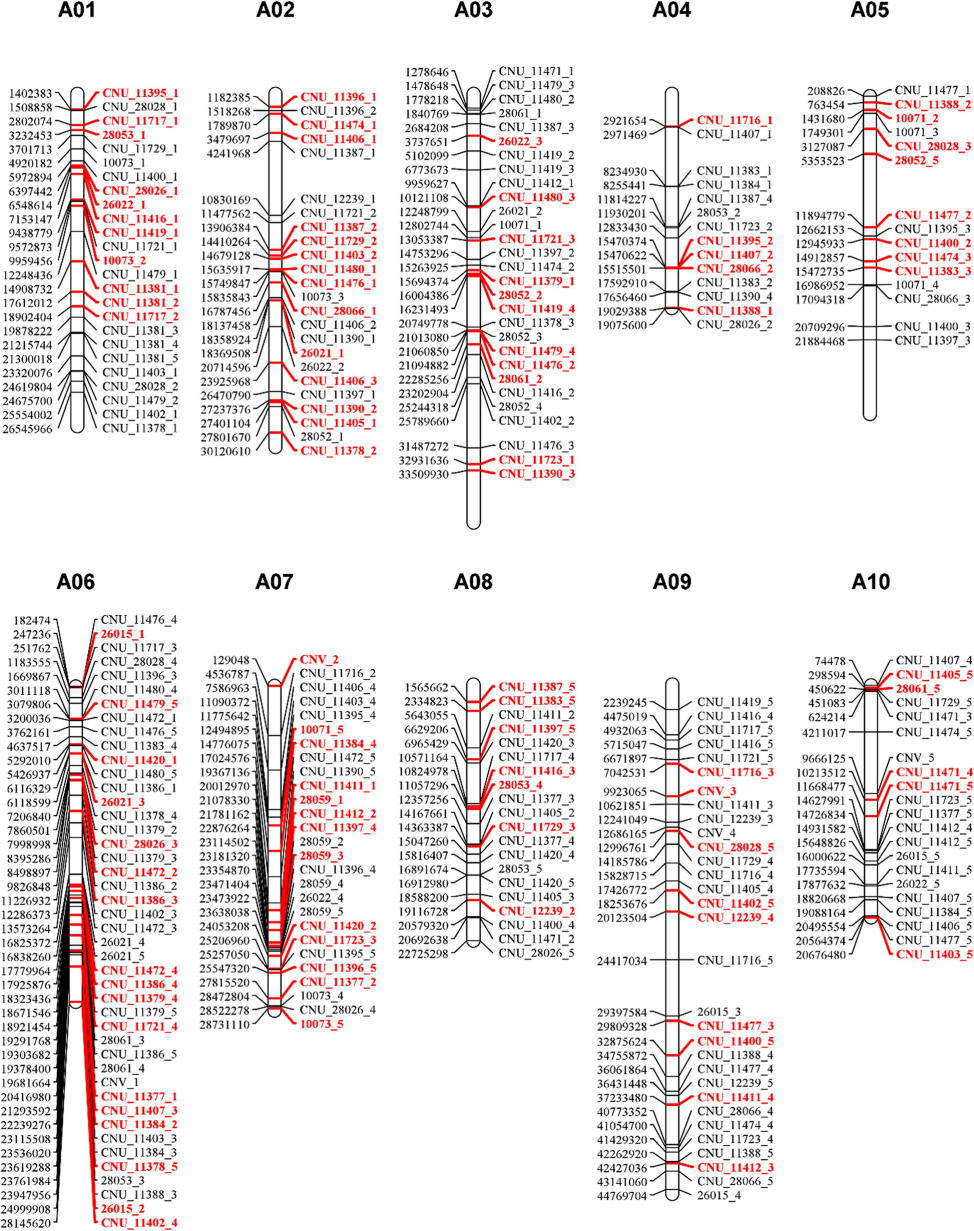
Genome distribution of accession-specific SNPs from the *Brassica rapa* core collection. The genomic positions of five accession-specific SNPs in each accession were investigated to develop KASP markers. (The marker positions with red color stand for SNPs used for KASP marker development)

Our next venture was to develop accession-specific KASP markers for assessment of hybrid seed purity. Five of the accession-specific SNP candidates that we identified as described above were selected from individual accessions for further analysis. Primer sites are important role in successful marker development, and we surveyed conserved flanking sequences of SNPs in our core collections (Fig. 3a). Flanking regions containing non-sequence sites, shown as N in the reference genome, were removed from the primer candidate sequences (Fig. 3b). Then, we selected five flanking sequences in each accession-specific SNP for further evaluation of KASP markers. It was necessary to consider the genomic position of the SNP in the development of a wide range of markers, as overlapping genomic positions among markers may lead to inefficiency or false positive results when assessing seed purity. To avoid this redundancy, we investigated the genomic positions of five candidate SNPs from individual accessions and selected the positions unique to the accessions (Fig. 4). In total, we selected two SNPs in each accession for validation of KASP markers (Supplementary Table 3). Many of the KASP markers that were in genic regions caused non-synonymous variation, although almost all accession-specific SNPs were detected in the flanking regions of genes (Table 2).

**Fig. 5:**
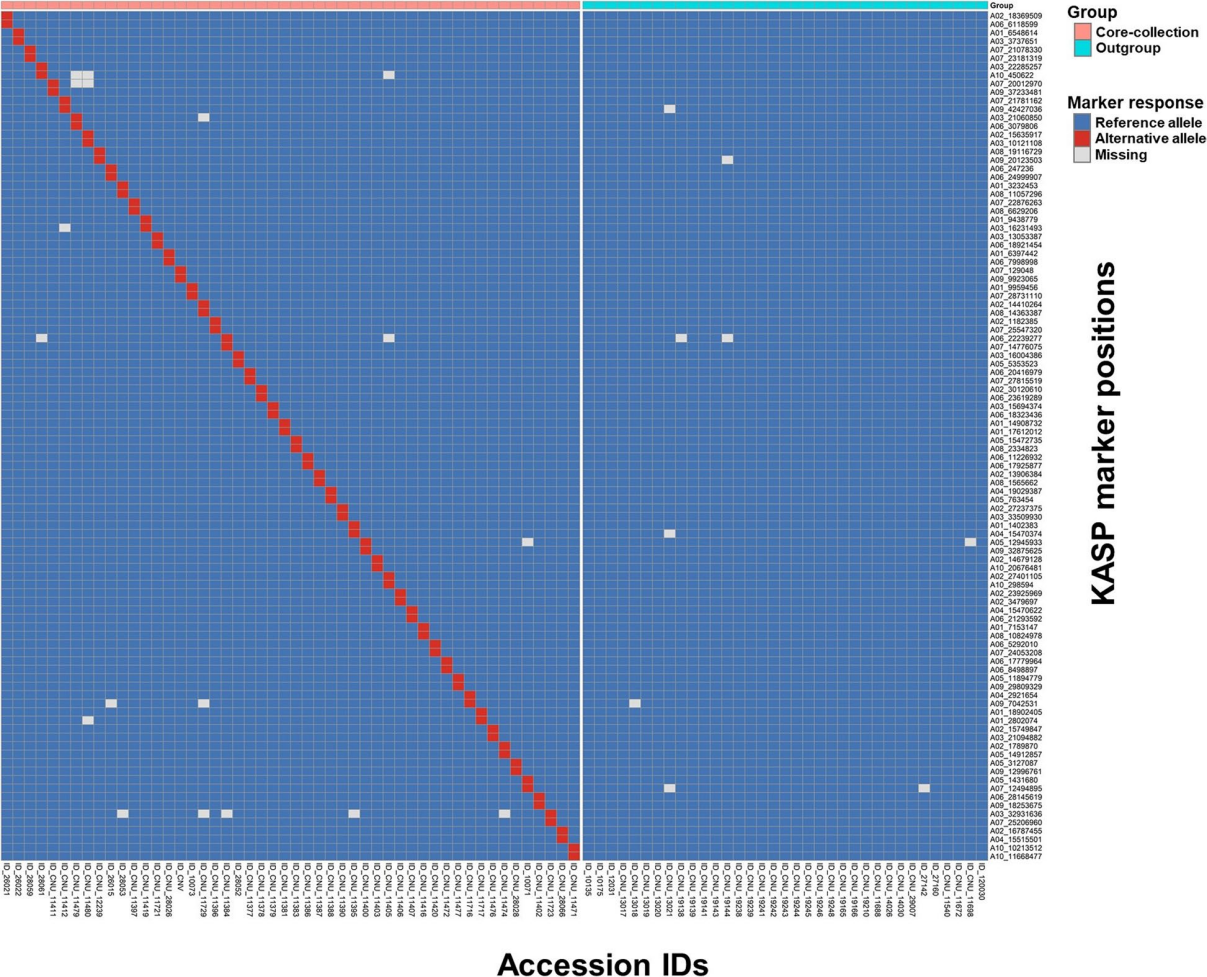
Validation of KASP markers using the *Brassica rapa* core collection, non-core collection, and commercial varieties. (Red bar on the top of heatmap stands for core collection, and blue bar stands for outgroup.)

Validation of KASP markers was carried out using 50 accessions from core collection and 35 from non-core collections, and 190 breeding lines provided by (Dayi International Seed Co.) for their applicability to a wide range of seed purity assessments (Fig. 5, Table 3, and Supplementary Data 2 and 3). Based on the results, we conclude that we successfully distinguished accession-specific markers in individual accessions in both the core collection and the outgroup (Fig. 5, Supplementary Fig. 10, and Supplementary Data 3). We suggest that accession-specific markers developed using a large amount of individual resequencing data can be used to assess seed purity from non-sequenced accessions or cultivars. The accession-specific markers developed here should be useful in a wide range of seed purity assessments in the *B. rapa* breeding and commercial seed production. We evaluated their ability to distinguish parental lines and F1 hybrids by testing two groups of parental lines and their F1 hybrid with the KASP marker A07_20012970. Results indicated that this marker successfully distinguished parental lines and the F1 (Supplementary Fig. 9). From these data, we suggest that KASP markers will be useful to plant breeders in assessing seed purity.

**Table 3:** Accession-specific single nucleotide polymorphisms (SNPs) that were identified in eight representative *B. rapa* accessions

Category	Accession ID	Chromosome	Position	REF	ALT	CNU_11479	CNU_11480	26,021	26,022	28,059	28,061	CNU_11411	CNU_11412
Non-pekinensis	CNU_11479	A03	21,060,850	A	C	CC	AA	AA	AA	AA	AA	AA	AA
		A06	3,079,806	A	G	GG	AA	AA	AA	AA	AA	AA	AA
	CNU_11480	A02	15,635,917	C	T	CC	TT	CC	CC	CC	CC	CC	CC
		A03	10,121,108	G	A	GG	AA	GG	GG	GG	GG	GG	GG
Chinese germplasm	26,021	A02	18,369,509	C	T	CC	CC	TT	CC	CC	CC	CC	CC
		A06	6,118,599	G	A	GG	GG	AA	GG	GG	GG	GG	GG
	26,022	A01	6,548,614	A	T	AA	AA	AA	TT	AA	AA	AA	AA
		A03	3,737,651	A	C	AA	AA	AA	CC	AA	AA	AA	AA
Japanese breeding	28,059	A07	21,078,330	G	T	GG	GG	GG	GG	TT	GG	GG	GG
		A07	23,181,319	G	T	GG	GG	GG	GG	TT	GG	GG	GG
	28,061	A03	22,285,257	G	A	GG	GG	GG	GG	GG	AA	GG	GG
		A10	450,622	A	C	AA	AA	AA	AA	AA	CC	AA	AA
Korean breeding	CNU_11411	A07	20,012,970	G	A	GG	GG	GG	GG	GG	GG	AA	GG
		A09	37,233,481	C	T	CC	CC	CC	CC	CC	CC	TT	CC
	CNU_11412	A07	21,781,162	C	G	CC	CC	CC	CC	CC	CC	CC	GG
		A09	42,427,036	C	G	CC	CC	CC	CC	CC	CC	CC	GG

*REF* Reference genome, *ALT* allele of reference genome and genetic variants, *CNU* Chungnam National University, a provider of 35 accessions used in this study

## Discussion

Molecular markers are promising tools to identify seed genotypes but have some limitations at present, as discussed above. With the advent of next-generation sequencing technology, construction of high-quality reference genomes and genetic information for many different cultivars and species has been generated. This information should provide the background necessary for the development of molecular markers that will provide accurate information and will be useful in a wide range of applications. These will include studying genetic variants in individual accessions, varieties, and large populations [15]. Reference genomes also provide useful detailed information on genetic variants such as gene structures, repetitive sequences, and accurate positions of various genetic features. This technology also applies to correlation analyses of phenotypes and may prove useful in analyses such as quantitative trait locus mapping and genome-wide association studies (GWAS) [16–18].

The marker screening step is used to select reliable markers among the candidate variants as part of the development of molecular markers for breeding. To develop KASP markers that distinguish different *B. rapa* genotypes, SNPs were identified from ten representative genotypes and selected by considering marker assay results such as reproducibility, missing rate, and genetic distribution [19]. However, these markers may not distinguish accessions that belong to the outgroup. To resolve this potential problem, we resequenced all accessions in our population, which covered different geographic origins (Supplementary Data 1). Accession-specific SNPs were identified and verified by Sanger sequencing (Fig. 2 and Supplementary Figs. 2, 3, 4, 5, 6 and 7) and accession-specific KASP markers were developed and validated with the outgroup. We conclude that the accession-specific KASP markers identified here are reliable and applicable to a wide range of genotypes.

In the current study, we identified SNPs in the *B. rapa* core collection with genome resequencing (Fig. 1). From the examination of accession-specific genetic variants, we identified 4,925,742 SNPs in 50 accessions among these, we identified 2,925 SNPs that were specific to a single accession (Table 1). Most genetic variants were detected in flanking regions of genes, but KASP markers were developed from SNPs that caused non-synonymous variations and were in genic regions. Conservation of the genic regions may have maintained the function of the genes, accounting for our observation that the ratio of conserved sequences was greater than for the other regions. The non-synonymous mutations might be involved in phenotypic or morphological differences among accessions and should be useful in investigation of trait-associated genes or markers associated with traits.

Until quite recently, molecular markers had not been developed for crops or cultivars, and those that are available have limited application. We developed molecular markers using the core collection of *B. rapa*, in part, to address this problem: we sought to develop markers, considering conserved sequence for primer sites, for a wide range of applications. (Fig. 3). Furthermore, we investigated genomic positions of accession-specific markers to avoid overlapping of the genomic positions of KASP markers (Fig. 4). In total, 100 accession-specific markers were developed as accession-specific KASP markers. Based on the results of our validation experiments, we are confident that we successfully distinguished the accession-specific markers in individual accessions in test populations from non-core or commercial cultivars (Fig. 5 and Supplementary Fig. 9). However, we did not develop enough KASP markers to guarantee their wide-ranging ability to evaluate seed purity during breeding or seed production. To enhance this possibility, more accession-specific KASP markers will be developed from 50 accessions and resequencing analysis with non-sequenced core collection will be conducted. These data suggested that seed assessments using KASP markers will contribute to *B. rapa* breeding by reducing breeding cycle time or seed production by maintaining high purity.

## Conclusions

In this study, we present efficient methods for developing KASP markers to distinguish individuals from a mixture of breeding lines and germplasms. We have employed the resequencing data of Chinese cabbage (*B. rapa* spp. *pekinensis*) in the development of KASP markers. We show that the accession-specific SNPs identified by NGS data pipelines are feasible targets for the development of KASP markers. We anticipate that the KASP markers developed here will be applicable to assessment of seed purity in a wide variety of situations, and will be applicable to core collections, other non-sequenced accessions, and commercial cultivars. These markers should also prove useful to breeding programs of *B. rapa,* facilitating the essential maintenance of pure parental lines. Furthermore, the non-synonymous mutations detected here should aid investigations of genes or markers associated with traits and in functional studies of genes. This study should facilitate marker development for assessment of the seed purity of commercial F1 seed samples whether or not they were produced by unintended crossing.

## Methods

### Plant materials

We wished to develop accession-specific KASP markers. To this end, 50 accessions of *Brassica rapa* core collections [20] were used in whole genome resequencing analysis. These accessions were characterized as inbred lines or doubled haploid lines. Thirty-five accessions (F1 hybrids and germplasm) donated by Chungnam National University (CNU) were used as the control panel showing high heterozygosity to validate the KASP markers. The reliability of developed KASP markers was confirmed with 190 Chinese cabbage accessions provided by Korean seed company (Dayi International Seed Co.)

### Genome resequencing of core collection

Truseq Nano DNA libraries were constructed according to the manufacturer’s instructions. To generate a large 550 bp insert, 100 ng or 200 ng of high molecular weight genomic DNAwas sheared with the Covaris S2 system to yield DNA fragments. Blunt-ended DNA fragments were generated with a combination of fill-in reactions and exonuclease activity. A single base A was then added to the blunt ends of each strand in preparation for ligation to the indexed adapters. Each adapter contained a single base T overhang for ligating the adapter to the A-tailed fragmented DNA. Ligated products were amplified with reduced-bias PCR. The quality of the amplified libraries was verified with capillary electrophoresis (Bioanalyzer, Agilent). After QPCR using SYBR Green PCR Master Mix (Applied Biosystems), we combined index-tagged libraries in equimolar amounts in the pool. Whole genome resequencing was performed with an Illumina NovaSeq 6000 system, following the protocols provided for 2 × 100 sequencing.

### Identification of genetic variants

The FastQC (v.0.11.3) program was used to assess quality and to detect adaptor sequences of reads (https://www.bioinformatics.babraham.ac.uk/projects/fastqc/). Adaptor sequences and low-quality reads were filtered using Trimmomatic (ver 0.36) with the parameter ILLUMINACLIP:TruSeq3-PE-2.fa:2:30:10 SLIDINGWINDOW:4:20 TRAILING:20 MINLEN:75 [21]. Then, the filtered reads were aligned to the *B. rapa* reference genome (ver 3.0) [11] with Burrows Wheel Aligner (BWA) (ver 0.1.17), using the default parameter [22]. These results (*.sam) were converted to bam files using SAMtools (ver 1.9) [23] and low-quality reads (mapping quality < 30) were removed. We also removed reads duplicated by PCR, with MarkDuplicate in Picard tools (ver 2.21.1) (http://broadinstitute.github.io/picard/). To detect InDels, InDels of the reference genomes were detected with RealignerTargetCreator in GATK (ver 3.7) [24] and reads mapped InDels were re-aligned with IndelRealigner. We detected and filtered SNPs(read depth > 3, genotype quality > 30, homozygous allele only) with BCFtools (ver 1.9) [25]. Possible SNP positions in the core collection were identified by conducting joint variant calling for all possible SNP positions in each accession. Multiple allelic positions and low-depth genotypes (read depth < 3) were filtered with VCFtools (ver 0.1.13) [26].

### Construction of a pipeline for accession-specific variants calling

We selected positions of SNPs that had homozygous alternative alleles for one accession in the population variant call format (vcf) file as accession-specific variants by an in-house perl script. To select KASP marker candidates from the accession-specific SNPs we had identified, we developed filtering steps, considering multiple properties of SNPs (Supplementary Fig. 1). To reduce the possibility of primer amplification for multiple loci, target sequence redundancy in the *B. rapa* genome was estimated with the megablast task of BlastN [27], and we detected 501 bp sequences harboring accession-specific SNPs. Accession-specific SNPs without flanking sequence redundancy were selected for KASP primer design. Also, accession-specific SNPs with flanking sequence overlapping predicted repeat sequences were filtered out with a gff file provided by the *B. rapa* reference genome ver 3.0. Accession-specific variants of the exon region were given priority for KASP primer design after SNP annotation by snpEFF [28]. The candidates for Sanger sequencing were determined by selecting the top two SNPs of read depth and genotype quality in each accession from four groups with different geographical origins. Representative data from each accession are shown (Fig. 2 and Supplementary Figs. 2, 3, 4, 5, 6, 7 and 8).

### Construction of pipeline for KASP marker development

We sought to minimize the failure of primer amplification that resulted from insertion or deletion on the marker target sites (Fig. 3a). This led us to develop a pipeline for producing KASP candidate sequences for accession-specific variants. The pipeline we developed generates flanking region sequences that harbor accession-specific variants from bam files of each accession and aligns them based on the reference genome sequence with ClustalW (-OUTPUT = CLUSTAL -TYPE = DNA -GAPOPEN = 10 -ENDGAPS -GAPDIST = 0.05) [29]. In the pipeline, the proportion of missing or alternative alleles from all of the aligned positions were evaluated and consensus sequences masking variable positions (non-reference allele for positions > 10%) with N were generated (Fig. 3b). Accession-specific variants located at 251 bp on the consensus sequences were used directly for the KASP primer designed by the manufacture’s protocol (LGC Genomics, UK).

### Evaluation and application of KASP markers

The KASP markers were validated with the Nexar system (LGC Douglas Scientific, Alexandria, USA) at the Seed Industry Promotion Center of the Foundation of Agricultural Technology Commercialization and Transfer (Gimje, Korea). An aliquot (0.8 L) of 2 × Master mix, 0.02 L of 72 × KASP assay mix (both from LGC Genomics), and 5 ng genomic DNA template from the 50 target *B. rapa* accessions of KASP markers and 35 *B. rapa* accessions in the outgroup were mixed into 1.6 L of KASP reaction mixture in a 384-well Array Tape. We ran duplicate reactions, and included non-template controls in each run. KASP amplification was performed with the following thermal cycling profile: 15 min at 94℃, a touchdown phase of 10 cycles at 94℃ for 20 s and at 61℃-55℃, in which the temperature decreased by 0.6℃ per cycle, for 60 s, and 26 cycles at 94℃ for 20 s and 55℃ for 60 s (first PCR stage). Next, recycling was performed with three cycles of 94℃ for 20 s and 57℃ for 60 s (second PCR stage). Recycling was performed twice, and the fluorescence value was used for KASP genotyping after PCR amplification.

## Supplementary Information

**Additional file 1.**

**Additional file 2.**

**Additional file 3.**

## Data Availability

The datasets have been deposited at NCBI under BioProject number PRJNA787013. The whole genome resequencing data for 50 *B. rapa* accessions are available through the NCBI Sequence Read Archive (https://www.ncbi.nlm.nih.gov/sra/SRP354760) with Bam file format.
