# Supplementary material for: Identification of accession-specific variants and development of KASP markers for assessing the genetic makeup of Brassica rapa seeds
Source: BMC Genomics. 2022 Apr 25;23:326. doi: 10.1186/s12864-022-08567-9 (PMC9036802; doi:10.1186/s12864-022-08567-9)
Supplement: Supplementary file 2 — Additional file 2. [file 12864_2022_8567_MOESM2_ESM.docx]

Supplementary Material


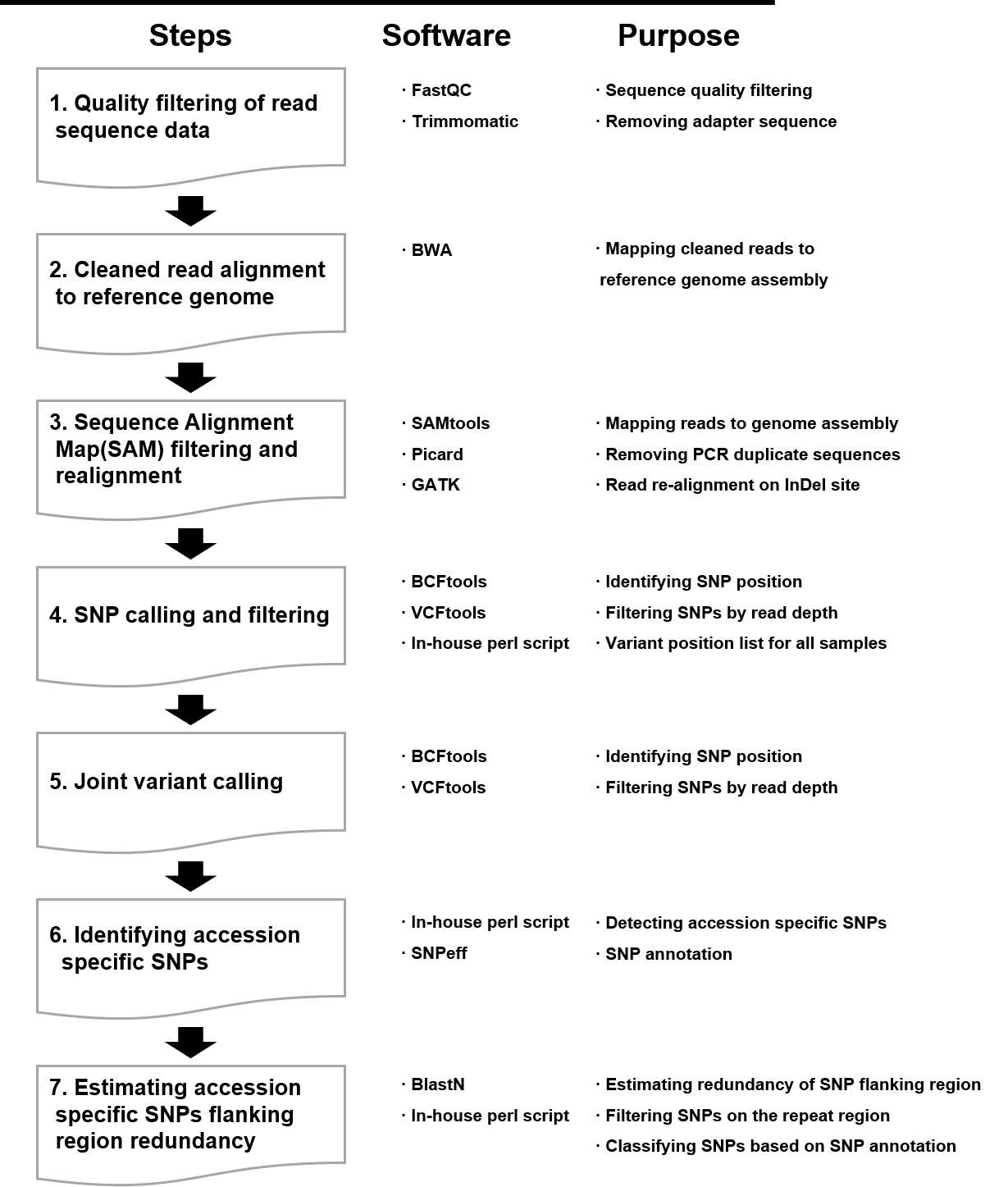


**Supplementary Figure 1.** Construction of pipeline for developing accession-specific markers


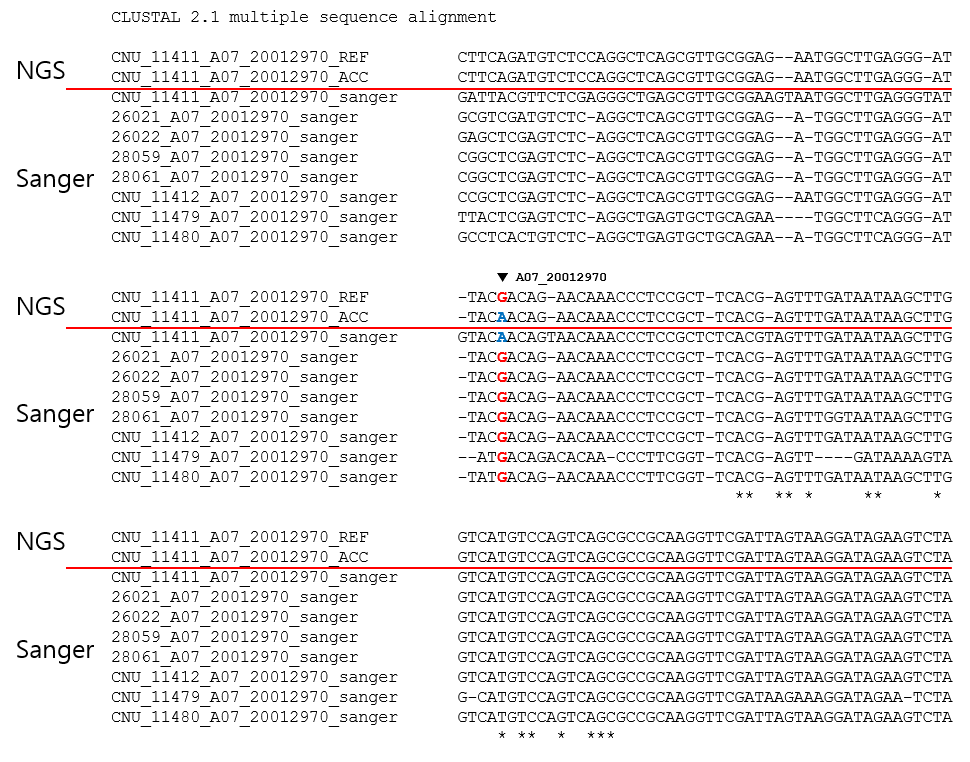


**Supplementary Figure 2.** Validation of accession-specific SNP (20,012,970 in chromosome 7) in accession CNU_11411 using *B. rapa* reference genome (ver 3.0). REF, ACC, and Sanger stands for reference genome, resequencing result of individual accessions, and sanger sequencing result, respectively.


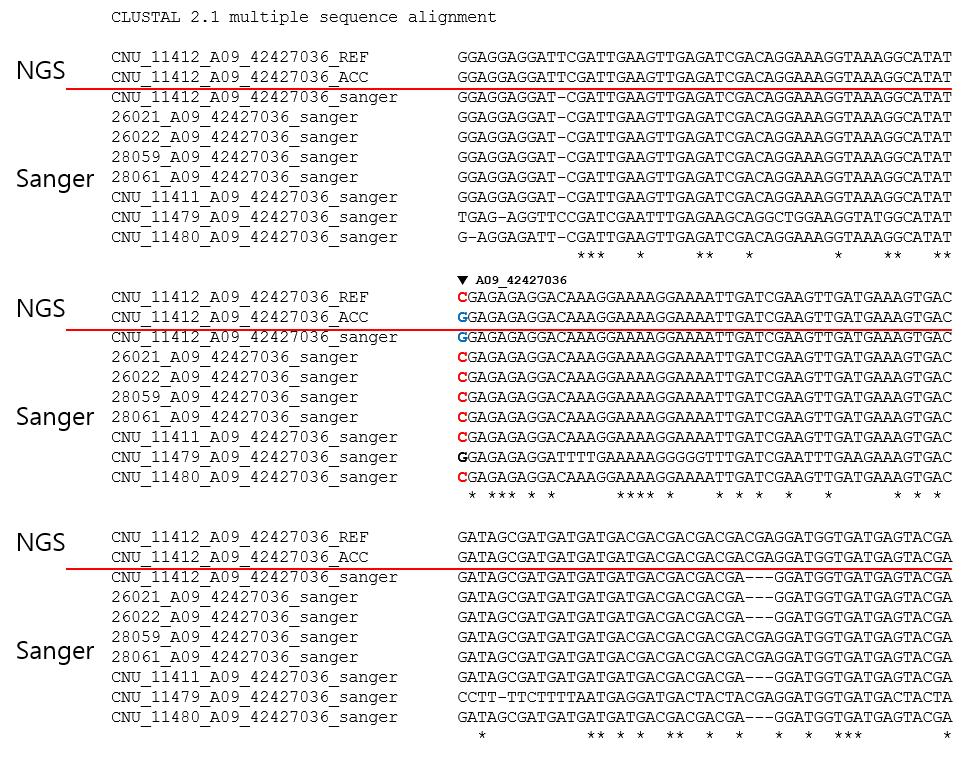


**Supplementary Figure 3.** Validation of accession-specific SNP (42,427,036 in chromosome 9) in accession CNU_11412 using *B. rapa* reference genome (ver 3.0). REF, ACC, and Sanger stands for reference genome, resequencing result of individual accessions, and sanger sequencing result, respectively.


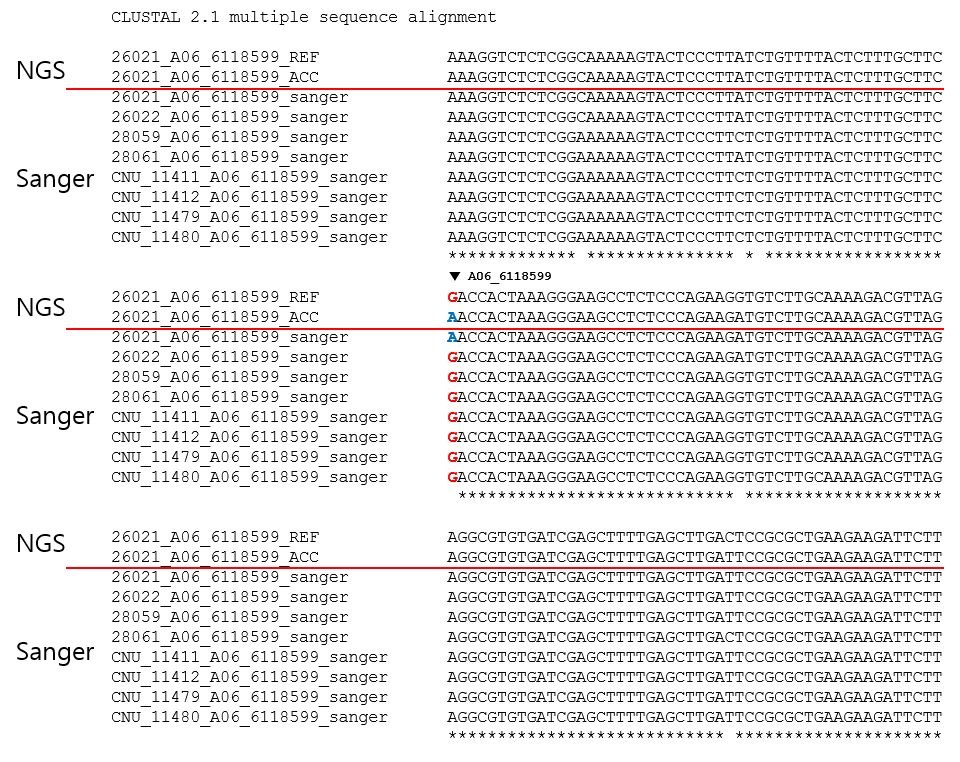


**Supplementary Figure 4.** Validation of accession-specific SNP (6,118,599 in chromosome 6) in accession 26021 using *B. rapa* reference genome (ver 3.0). REF, ACC, and Sanger stands for reference genome, resequencing result of individual accessions, and sanger sequencing result, respectively.


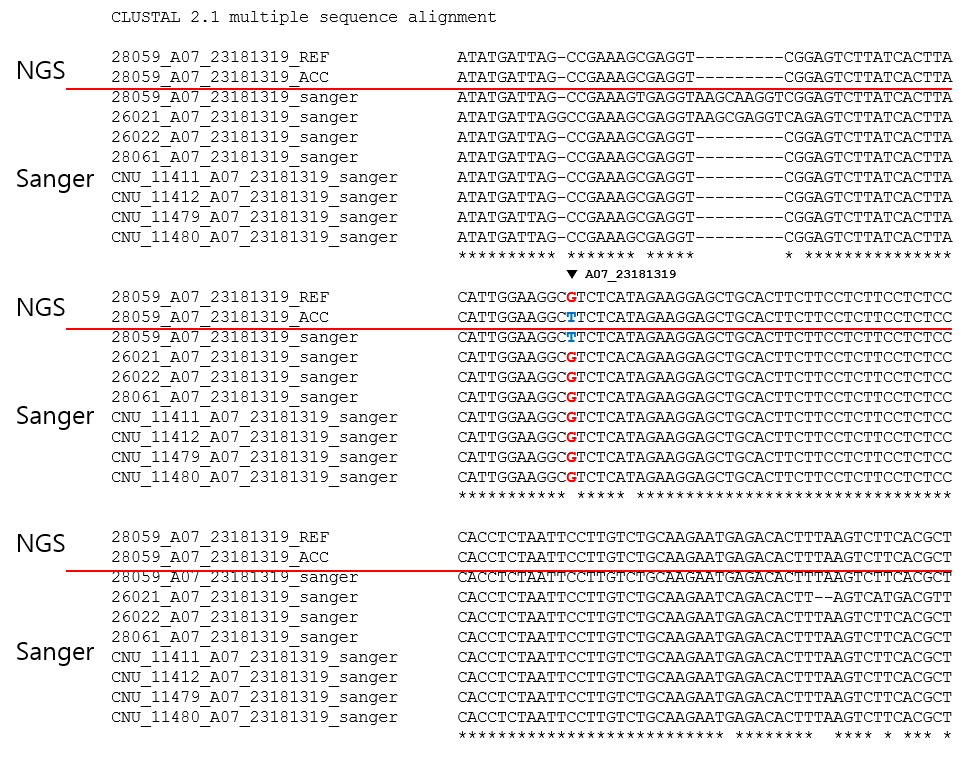


**Supplementary Figure 5.** Validation of accession-specific SNP (23,181,319 in chromosome 7) in accession 28059 using *B. rapa* reference genome (ver 3.0). REF, ACC, and Sanger stands for reference genome, resequencing result of individual accessions, and sanger sequencing result, respectively.


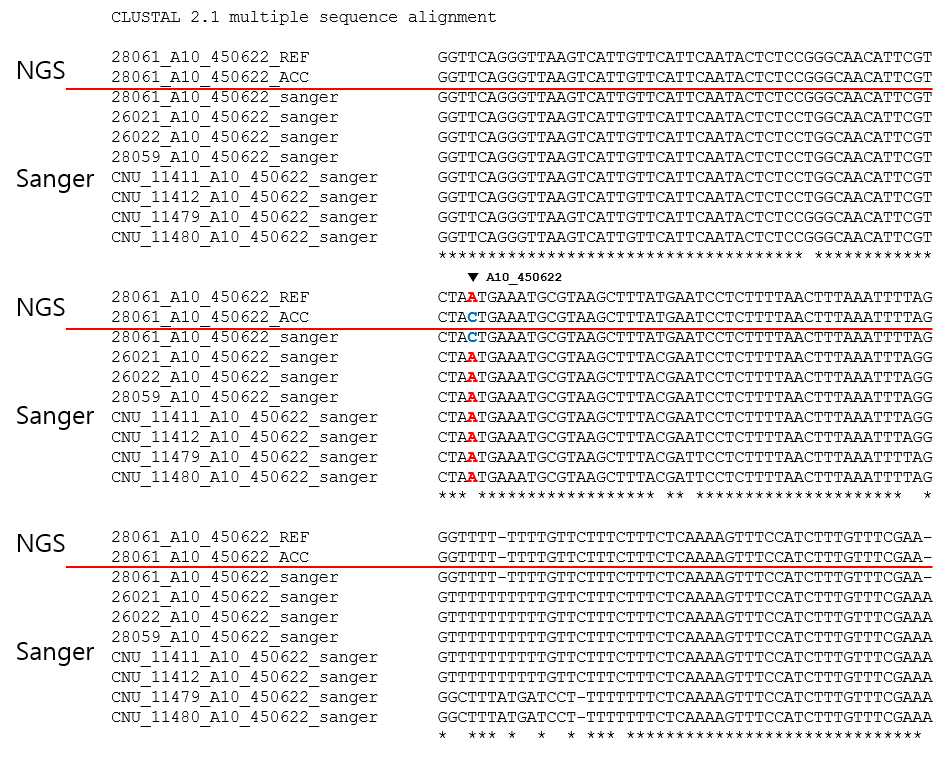


**Supplementary Figure 6.** Validation of accession-specific SNP (450,622 in chromosome 10) in accession 28061 using *B. rapa* reference genome (ver 3.0). REF, ACC, and Sanger stands for reference genome, resequencing result of individual accessions, and sanger sequencing result, respectively.


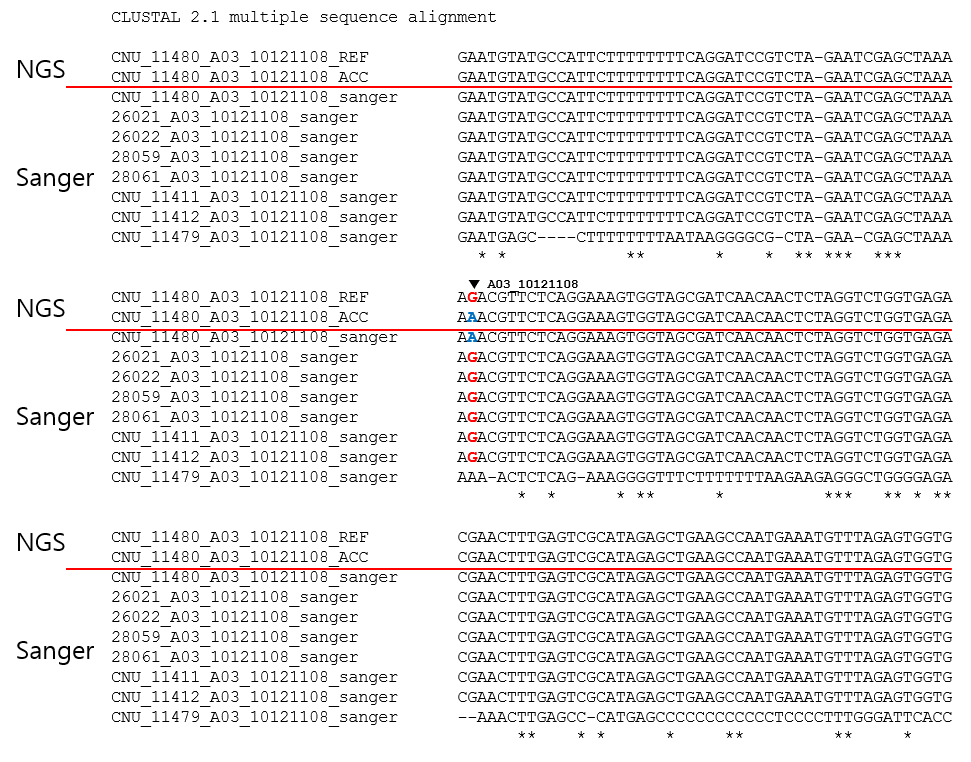


**Supplementary Figure 7.** Validation of accession-specific SNP (10,121,108 in chromosome 3) in accession CNU_11480 using *B. rapa* reference genome (ver 3.0). REF, ACC, and Sanger stands for reference genome, resequencing result of individual accessions, and sanger sequencing result, respectively.


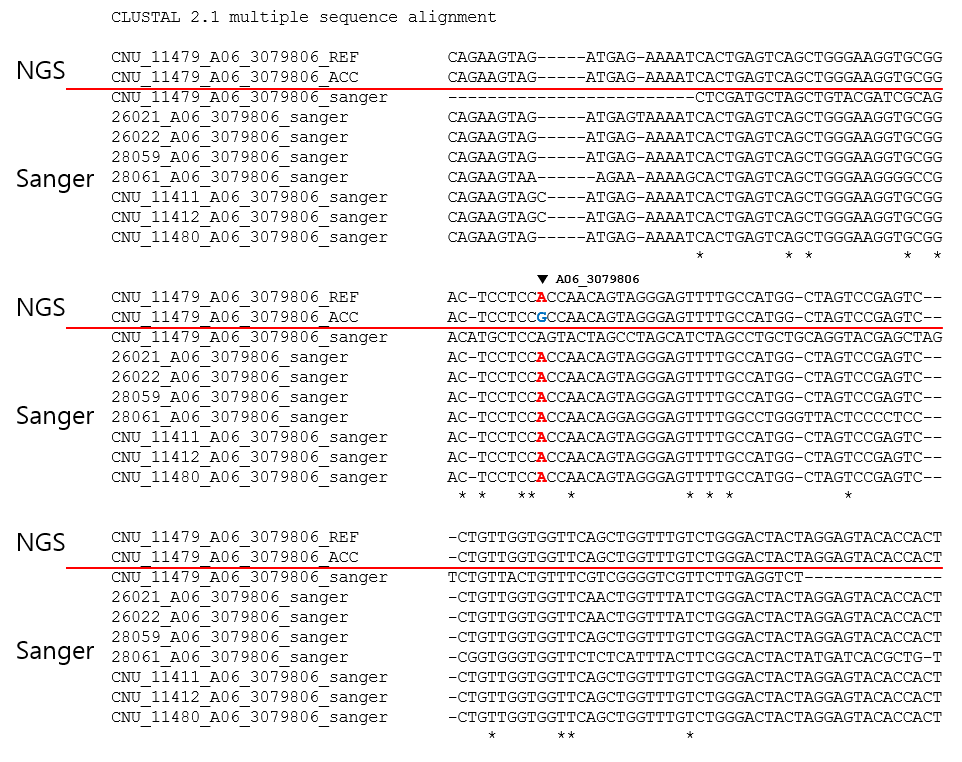


**Supplementary Figure 8.** Validation of accession-specific SNP (3,079,806 in chromosome 6) in accession CNU_11479 using *B. rapa* reference genome (ver 3.0). REF, ACC, and Sanger stands for reference genome, resequencing result of individual accessions, and sanger sequencing result, respectively.


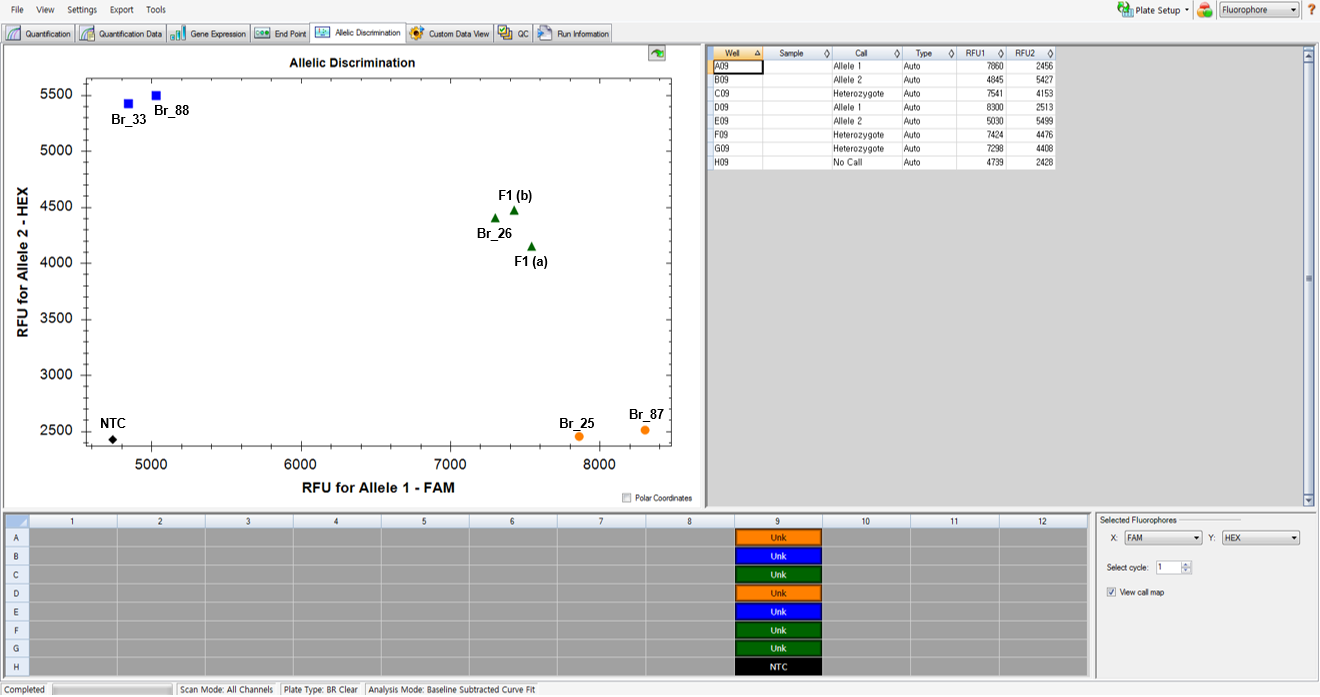


**Supplementary Figure 9.** The result image of KASP marker assay for F1 hybrids and parental lines. F1(a) is a F1 hybrid between Br_33 and Br_25. F1(b) is a F1 hybrid between Br_87 and Br_88. (The Br_26 is determined as heterozygous allele for the KASP marker.)


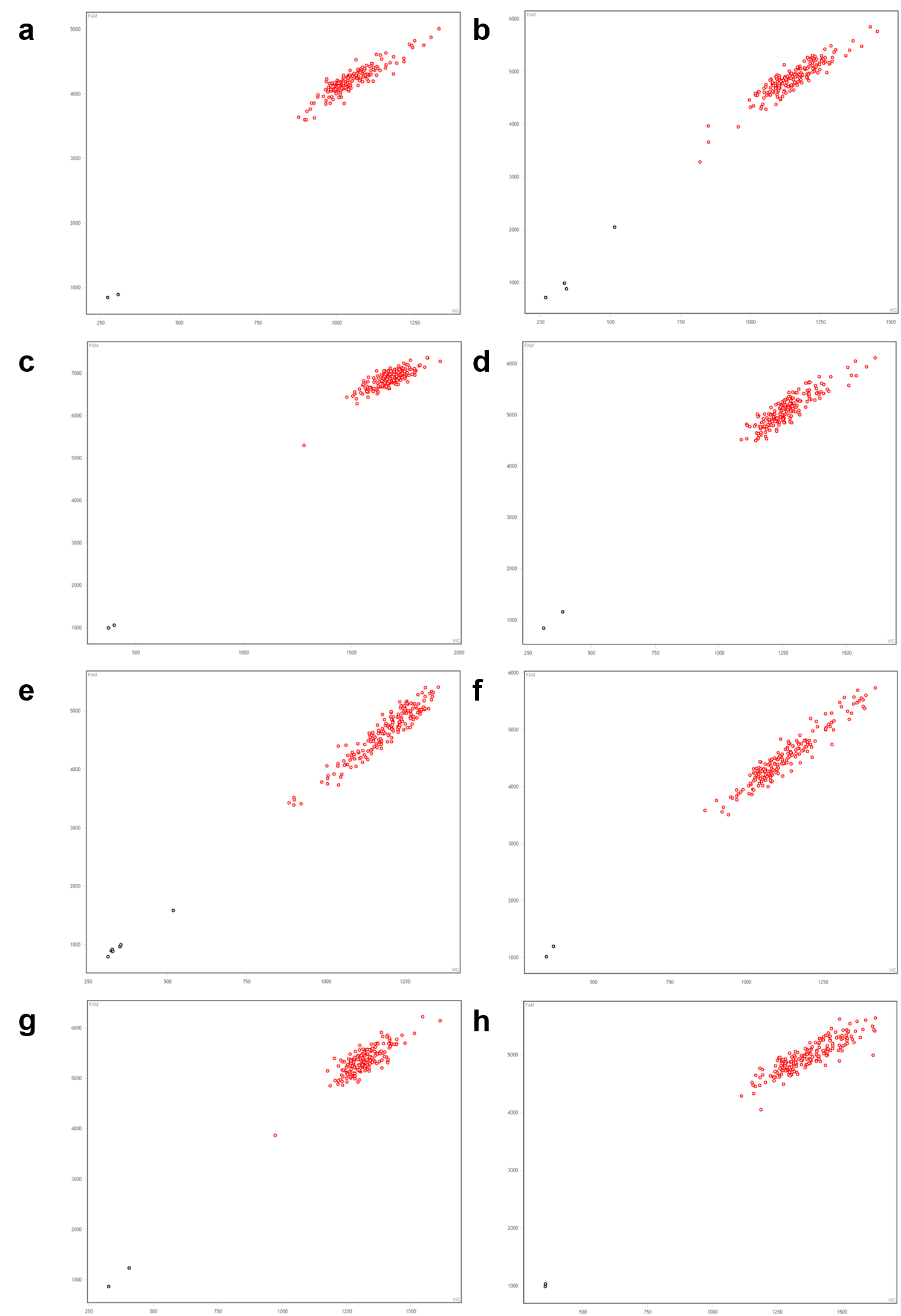


**Supplementary Figure 10.** The result image of KASP marker assay for 190 breeding lines for eight KASP markers. a) The result of A02_18369509 (Target accession: 26021), b) The result of A01_6548614 (Target accession: 26022), c) The result of A07_21078330 (Target accession: 28059), d) The result of A03_22285257 (Target accession: 28061), e) The result of A09_37233481 (Target accession: CNU_11411), f) The result of A07_21781162 (Target accession: CNU_11412), g) The result of A06_3079806 (Target accession: CNU_11479), h) The result of A02_15635917 (Target accession: CNU_11480)
